# Supplementary material for: A Review on Bioactive Anthraquinone and Derivatives as the Regulators for ROS
Source: Molecules. 2023 Dec 17;28(24):8139. doi: 10.3390/molecules28248139 (PMC10745977; doi:10.3390/molecules28248139)
Supplement: Supplementary file 1 [file molecules-28-08139-s001.zip › molecules-2729236-supplementary.pdf]

Supplementary Material

A Review on Bioactive Anthraquinone and Derivatives as the Regulators for ROS

Lihua Zhao [1] and Lin Zheng[2,*]

[1] Tianjin Renai College, Tianjin 301636, China; huazi.95@163.com
[2] College of Pharmaceutical Engineering of Traditional Chinese Medicine, Tianjin University of Traditional Chinese Medicine, Tianjin 301617, China
* Correspondence: zhenglin@tjutcm.edu.cn

Contents

Figure S1. The illustration of ROS formation. The symbols and abbreviations are as follows: $^{\bullet}OH$, hydroxyl radical; $O_2^{\bullet-}$, superoxide radical; H_2O_2, hydrogen peroxide; OH^-, hydroxide anion; SOD, superoxide dismutase; CAT, catalase; GPX, glutathione peroxidase; GSH, reduced glutathione; GSSG, oxidized glutathione; GSR, glutathione reductase; GST, glutathione *S*-transferase.

Figure S2. Redox cycling of quinones and the effect of the different components. One-electron reduction ($1e^-$) of the semiquinone undergoes aerobic oxidation [O] to generate $O_2^{\bullet-}$ and the parent quinone. Under anaerobic conditions, the semiquinone could undergo a disproportionation reaction yielding quinone and the corresponding hydroquinone. Two-electron reduction ($2e^-$) of quinone produces hydroquinone, which may undergo a compounding reaction with quinone to produce semiquinone that is oxidized to semiquinone and $O_2^{\bullet-}$, producing reactive alkylating agents and/or is excreted from the body. The semiquinones and $O_2^{\bullet-}$ produced by the process eventually lead to oxidative stress and cell damage.

Table S1. HOMO, LUMO and IP energies calculated at the B3LYP/6-311++G** level of theory, and scavenging activity on DPPH radical.

Table S2. The BDE value of free radical species (1-OH, 3-OH, and 8-OH) of emodin (**1**).

Table S3. ΔBDE, ΔPDE, and ΔIP values of OH groups in purpurin (**10**) (relative to phenol).

Table S4. The pharmacokinetic parameters of anthraquinones in dogs.

Table S5. Radical scavenging activity of hydroxyanthraquinones.

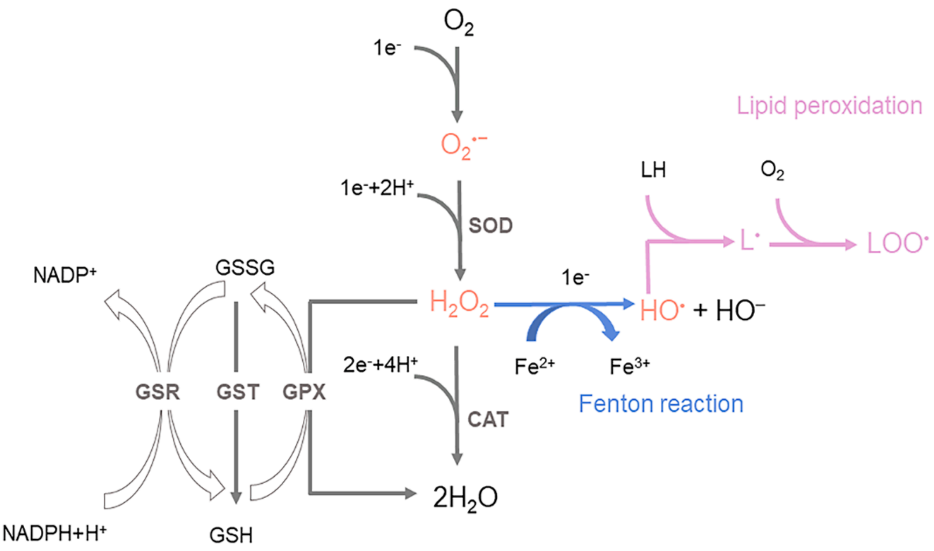

Figure S1. The illustration of ROS formation. The symbols and abbreviations are as follows: •OH, hydroxyl radical; $O_2^{\bullet-}$, superoxide radical; H_2O_2, hydrogen peroxide; OH^-, hydroxide anion; SOD, superoxide dismutase; CAT, catalase; GPX, glutathione peroxidase; GSH, reduced glutathione; GSSG, oxidized glutathione; GSR, glutathione reductase; GST, glutathione *S*-transferase.

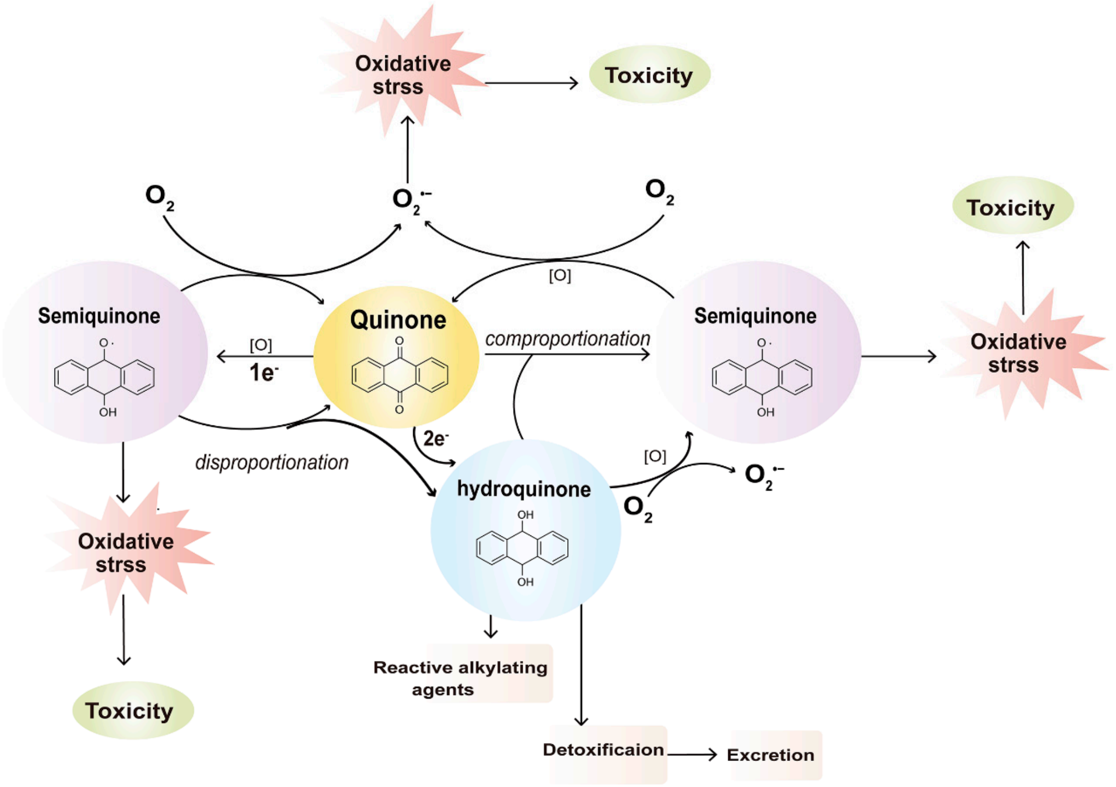

Figure S2. Redox cycling of quinones and the effect of the different components. One-electron reduction (1e-) of the semiquinone undergoes aerobic oxidation [O] to generate $O_2^{\bullet-}$ and the parent quinone. Under anaerobic conditions, the semiquinone could undergo a disproportionation reaction yielding quinone and the corresponding hydroquinone. Two-electron reduction (2e-) of quinone produces hydroquinone, which may undergo a compounding reaction with quinone to produce semiquinone that is oxidized to semiquinone and $O_2^{\bullet-}$, producing reactive alkylating agents and/or is excreted from the body. The semiquinones and $O_2^{\bullet-}$ produced by the process eventually lead to oxidative stress and cell damage.

Table S1. HOMO, LUMO and IP energies calculated at the B3LYP/6-311++G** level of theory, and scavenging activity on DPPH radical.

**Comp.**	**Structure**	E^{HOMO} **(eV)**	E^{LUMO} **(eV)**	E^{gap} **(eV)**	**IP(O[a]) (eV)**	**IP(E[b]) (eV)**	**Scavenging activity on DPPH[c] (μM)**
**Aloe-emodin (2)**	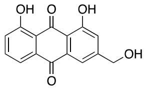	-6.6	-3.11	3.48	6.6	0.29	222 [150]
**Aloesone**	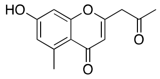	-6.67	-1.06	5.07	6.67	0.30	351 [150]
**Isoeleutheol**	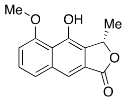	-5.97	-1.88	4.09	5.97	0.23	217 [151]

HOMO, highest occupied molecular orbital; LUMO, lowest unoccupied molecular orbital; IP, ionization potential; O[a], orbital consideration (orbital-vertical); *E*[b], energy consideration (energy-vertical); DPPH[c], Trolox equivalents.

Table S2. The BDE value of free radical species (1-OH, 3-OH, and 8-OH) of emodin (**1**).

	**Calculating method**	**BDE (kJ·mol^{-1})**
**1-OH**	B3LYP/6311++ G**	412.25
**3-OH**	B3LYP/6311++ G**	371.12
**8-OH**	B3LYP/6311++ G**	408.57

BDE: bond dissociation enthalpy.

Table S3. ΔBDE, ΔPDE, and ΔIP values of OH groups in purpurin (**10**) (relative to phenol).

	**ΔBDE (kJ/mol)**			**ΔPDE (kJ/mol)**			**ΔIP (kJ/mol)**
	1-OH	2-OH	4-OH	1-OH	2-OH	4-OH	
P_{gas}	-16.85	-25.39	-47.81	84.51	102.61	29.66	-51.81
P_{pcm}	1.32	-10.67	-27.04	-223.08	-231.83	-198.48	-10.65

All theoretical values refer to phenol calculated with the same method.
BDE, bond dissociation enthalpy; PDE, proton dissociation enthalpy; IP, ionization potential; pcm, polarizable continuum solvation mode.

Table S4. The pharmacokinetic parameters of anthraquinones in dogs (Feng et al., 2014; Wang et al., 2021).

**Comp.**	C_{max} **(μg/ml)**	T_{max} **(h)**	$AUC_{0-\infty}$ **(mg h/l)**
**Emodin (1)**	0.27–0.48	0.75–1.42	1.38–4.05
**Aloe-emodin (2)**	0.03–0.45	0.75–1.55	0.35–1.61
**Rhein (3)**	1.44–3.39	0.71–1.50	4.24–35.1

**Physcion (4)**	0.03	2.00	0.41
**Chrysophanol (5)**	0.03–0.30	1.00–2.00	0.43–0.83

C_{max}, peak concentration; T_{max}, peak time; AUC, area under the curve.

Table S5. Radical scavenging activity of hydroxyanthraquinones (Cai et al., 2004).

Hydroxyanthraquinones	Structure	Equivalent antioxidant activity values
**1**	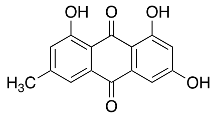	0.172 ± 0.002 mM
**2**	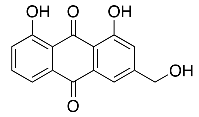	0.173 ± 0.001 mM
**3**	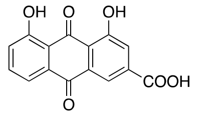	0.174 ± 0.001 mM
**4**	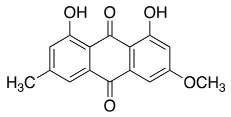	0.171 ± 0.002 mM
**5**	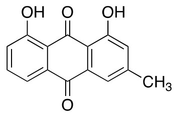	0.170 ± 0.001 mM
**10**	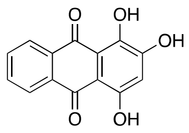	1.680 ± 0.009 mM
**11**	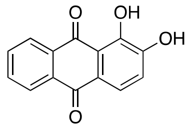	1.019 ± 0.008 mM
**12**	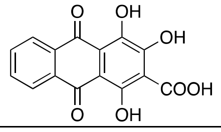	1.216 ± 0.011 mM
